# Study on the correlation between ventricular arrhythmia and heart rate variability in patients with coronary heart disease

**DOI:** 10.3389/fcvm.2026.1755369

**Published:** 2026-07-15

**Authors:** Xin Guan, Zhiyue Jia, Bing Yang

**Affiliations:** Electrocardiogram Room, Shanxi Provincial People’s Hospital, Taiyuan, Shanxi, China

**Keywords:** coronary heart disease, electrocardiogram, heart rate, linear regression, risk factor, ventricular arrhythmia

## Abstract

**Background:**

Ventricular arrhythmia (VA) is a common complication in patients with coronary heart disease (CHD), and heart rate variability (HRV) is considered a potential marker for VA risk.

**Aims:**

This study aimed to analyse the correlation between HRV and VA in patients with CHD.

**Methods:**

From January 2020 to July 2024, patients with CHD who were treated at our hospital were divided into two groups, based on the presence of VA: theVA group (VA present) and the control group (no VA). Heart rate variability indices were measured using 24-hour dynamic electrocardiogram monitoring. The HRV parameters included the standard deviation of sinus R-R (N-N) interval (SDNN), the standard deviation of sinus R-R (N-N) mean value every 5 min (SDANN), the root mean square of adjacent RR interval difference (rMSSD), and the percentage of the number of adjacent R-R interval differences >50 ms in the total number of sinus beats (PNN50). Clinical data were compared between groups, and linear regression was used to explore the relationship between HRV and VA.

**Results:**

The HRV indices SDNN, SDANN, rMSSD, and PNN50 were significantly lower in the VA group than in the non-VA group (*P* < 0.05). Linear regression analysis showed that SDANN, rMSSD and PNN50 were significantly associated with VA occurrence (*P* < 0.05). The linear equation derived was *Y* = 1.976 − 0.006 × *X*2 − 0.009 × *X*3 − 0.007 × *X*4.

**Conclusion:**

The HRV indices, particularly SDANN, rMSSD, and PNN50, were significantly associated with the occurrence of VA in patients with CHD and may serve as potential indicators for VA risk stratification.

## Graphical Abstract

**Figure:**
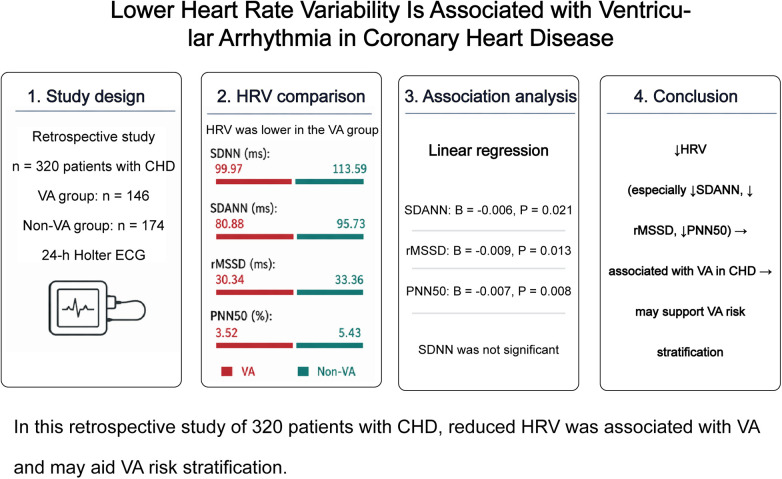

## 1 Introduction

Coronary atherosclerosis, also known as coronary heart disease (CHD), is a heart disease caused by atherosclerotic lesions in the coronary arteries. The process includes the formation, enlargement, and sclerosis of lipid plaques, which eventually leads to vascular stenosis or even complete occlusion (1). Middle-aged and elderly people are the high-risk population of CHD, and the disease is often accompanied by complications such as heart failure and arrhythmia. Coronary heart disease may lead to acute myocardial infarction and can even be life-threatening (2). Ventricular arrhythmia (VA), a common complication of patients with CHD, mainly manifests as palpitations, chest tightness, fatigue, and other symptoms. In severe cases, dyspnoea and syncope may also occur (3, 4). This study found that the occurrence of VA may aggravate the patient's condition and increase the mortality rate, especially in the case of poor drug compliance, low socioeconomic status, large blood glucose fluctuations, or electrical storms (5). Therefore, it is of great significance to provide patients with CHD and VA with a positive and effective diagnosis.

A dynamic electrocardiogram (ECG) is an important tool for evaluating cardiac function and autonomic nerve function by recording ECG signals over a long period of time. Its advantages include low cost, simple operation, and continuous 24-hour or longer monitoring of ECG activity; accordingly, it is widely used in clinical practice (6). Heart rate variability (HRV) is an index used to monitor changes in heartbeat interval, which reflects the ability of the autonomic nervous system to regulate the heart (7). Heart rate variability is closely related to a variety of diseases, notably cardiovascular disease, diabetes, and stroke (8–10). It has been reported that HRV plays an important role in judging the severity and prevention of CHD and can be used as a predictor of arrhythmia (11).

It is very important to explore the relationship between VA and HBV in CHD to reduce the mortality of patients with CHD. However, the current clinical research mainly focuses on arrhythmia, with few studies on VA and HBV. Therefore, the purpose of this study was to explore and analyse the relationship between HRV and VA, and to provide a reference for the treatment and prevention of clinical CHD.

## 2 Materials and methods

### 2.1 General information

This was a retrospective study. Ethics and medication management were addressed as follows: This study was conducted in accordance with the Declaration of Helsinki and approved by the Ethics Committee of Shanxi Provincial People's Hospital. Written informed consent was obtained from all participants. To minimise pharmacological influences on HRV assessment and VA detection, all patients included in the study were required to discontinue any medications that could affect heart rate and/or blood pressure for 3 days prior to 24-hour ambulatory ECG (Holter) monitoring, as specified by the research team protocol. Patients who were considered high-risk for medication discontinuation were not enrolled.

Only patients with complete eligible 24-hour ambulatory ECG (Holter) data suitable for HRV analysis were included according to the predefined eligibility criteria. During the study period, a total of 1,280 patients were hospitalized for coronary heart disease in our department, and 320 patients were finally included, accounting for 25.00% of all inpatients with coronary heart disease. They were divided into VA group (*n* = 146) and non-VA group (*n* = 174), based on whether VA was absent or present.

Among the 146 patients in theVA group, there were 89 cases of frequent ventricular premature beats, 35 cases of multi-source ventricular premature beats, and 22 cases of ventricular tachycardia (including 15 cases of non-sustained ventricular tachycardia and 7 cases of sustained ventricular tachycardia).

The inclusion criteria were as follows: (1) The enrolled patients met the diagnostic criteria for CHD in the “Chinese Guidelines for the Prevention of Cardiovascular Disease” (12) and were diagnosed by coronary angiography in the hospital. (2) The basic rhythm was a sinus rhythm, without atrioventricular block, intraventricular block, or an electrolyte disturbance. (3) Complete clinical data and clear imaging were available. (4) The baseline of the dynamic ECG was stable. (5) The participant agreed to participate in the study (with written informed consent obtained, as applicable). The exclusion criteria were as follows: (1) Patients with malignant tumours, abnormal thyroid function, severe abnormal liver and renal function, or any other important organ lesions were not enrolled. (2) Patients with an ectopic rhythm, or ‘New York Heart Association Functional Classification for Heart Failure Grades III–IV, or Killip classification grades III–IV, were excluded. (3) Patients who did not agree to participate in the study (refusal or inability to provide informed consent, as applicable) were excluded.

Ventricular arrhythmia diagnosis (13): VA was diagnosed based on 24-hour ambulatory ECG results. Ventricular arrhythmia includes frequent ventricular premature beats, multi-source ventricular premature beats, and ventricular tachycardia. Frequent ventricular premature beats refer to more than 6 ventricular premature beats in 1 min; multi-source ventricular premature beats are defined as more than 2 types of ventricular premature beats (excluding ventricular fusion waves formed by ventricular premature beats). Tachycardia describes a heart rate higher than 100 beats/min, 3 or more continuous or more spontaneous ventricular depolarisation activities, including persistent ventricular tachycardia (onset time greater than 30 s), and non-persistent ventricular tachycardia (onset time less than 30 s).

### 2.2 Detection method

All patients stopped taking heart rate or antihypertensive drugs 3 days before the test.

Before drug withdrawal, 217 cases of the 320 included patients received β-blocker treatment, and 49 cases received other heart rate-lowering drugs (non-dihydropyridine calcium channel blockers, etc.), accounting for 67.81% and 15.31% of the total sample respectively.

The 24 h dynamic ECG of the patients was detected by a 12-lead dynamic ECG/ambulatory blood pressure recorder and ECG machine (PageWriter TC20; Philips, Amsterdam, The Netherlands). The HRV time domain indexes were calculated as follows: standard deviation of sinus R-R (N-N) interval (SDNN), standard deviation of sinus R-R (N-N) mean value every 5 min (SDANN), the root mean square of adjacent RR interval difference (rMSSD), and the percentage of the number of adjacent R-R interval differences >50 ms in the total number of sinus beats (PNN50).

### 2.3 Observation indicators

The basic data of the enrolled patients were collected: age, gender (male, female), course of disease, and combined disease (e.g., hypertension, diabetes, hyperglycaemia, hyperlipidaemia). Cardiac function indexes were examined by colour Doppler ultrasound, including left ventricular ejection fraction (LVEF) and left ventricular end-diastolic diameter (LVEDD). The blood laboratory indexes of patients were measured by enzyme-linked immunosorbent assay, including total cholesterol (TC), triglyceride (TG), and low-density lipoprotein (LDL).

### 2.4 Statistical analysis

In this study, the data were analysed by SPSS 26.0 software. For continuous variables, the distribution was assessed for normality. The measurement data with a normal distribution were expressed as mean ± standard deviation (*x¯* ± *s*), and the *t*-test was used for between-group comparison. If a continuous variable did not meet the normality assumption, an appropriate non-parametric test would be considered. Categorical data, such as gender, were expressed as [*n* (%)], and the *χ*^2^ test was used for between-group comparison. The levels of HRV indexes were compared between the two groups, and their correlation with VA was analysed; *P* *<* 0.05 was considered statistically significant.

## 3 Results

### 3.1 Comparison of general data

**Figure 1:**
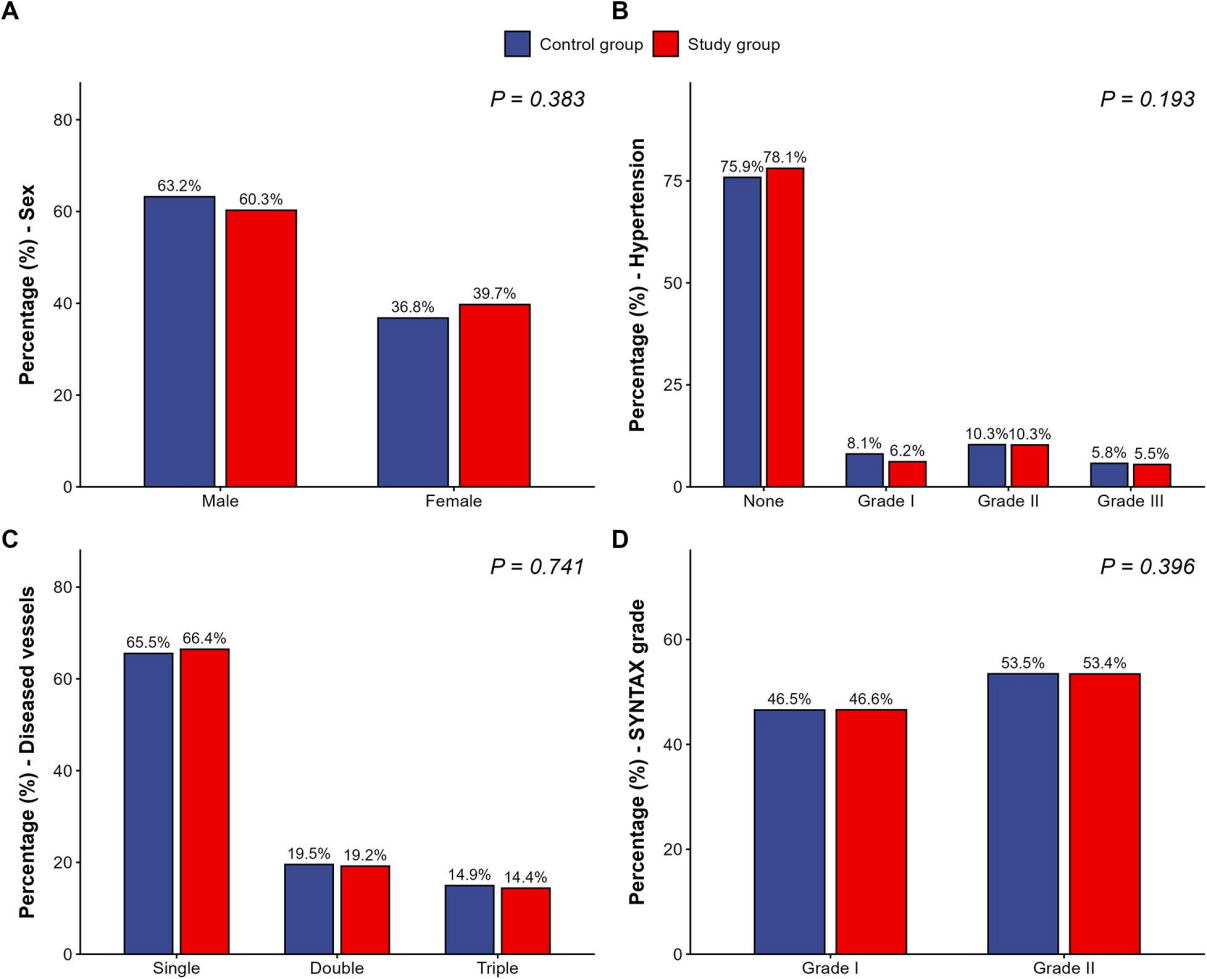
Baseline categorical characteristics of the enrolled patients. Grouped bar charts illustrate the distribution of **(A)** sex, **(B)** hypertension grade, **(C)** number of diseased vessels, and **(D)** SYNTAX grade between the non-VA group (*n* = 174) and the VA group (*n* = 146). Data are presented as percentages (%), with exact values labeled above each bar. *P*-values for the overall distribution differences between the two groups were calculated using the Chi-square test and are annotated in the top right corner of each panel.

A total of 320 patients with CHD were included in this study, with 146 in the VA group and 174 in the non-VA group. In the VA group, 88 patients were men and 58 were women, with a mean age of 60.73 ± 11.85 years and a disease duration of 4.85 ± 2.25 years. In the non-VA group, 110 patients were men and 64 were women, with a mean age of 61.94 ± 12.62 years and a disease duration of 4.66 ± 2.56 years. There was no significant difference in terms of gender, age, course of disease, hypertension, diabetes, hyperglycaemia, hyperlipidaemia, LVEF, LVEDD, TC, TG, and LDL between the two groups (*P* > 0.05), indicating that the two groups were comparable. In addition, the degree of coronary vessel disease (number of diseased branches: single-, double-, and triple-vessel disease) and SYNTAX score grade showed no significant differences between the two groups (both *P* > 0.05). The severity distribution of hypertension (Grade I–III) was also comparable between the two groups (*P* > 0.05) (see Table 1 and Figure 1).

**Table 1:** Comparison of baseline characteristics between the VA group and the non-VA group.

Index	VA group (*n* = 146)	Non-VA group (*n* = 174)	*χ*^2^/*t*	*P*
Age (year)	60.73 ± 11.85	61.94 ± 12.62	1.043	0.327
Gender (male, *n*/%)	88 (60.27)	110 (63.22)	3.802	0.383
Coure (year)	4.85 ± 2.25	4.66 ± 2.56	1.473	0.074
Hypertension (*n*/%)			1.792	0.193
Grade I	9 (6.16)	14 (8.05)		
Grade II	15 (10.27)	18 (10.34)		
Grade III	8 (5.48)	10 (5.75)		
Diabetes (*n*/%)	37 (25.34)	47 (27.01)	2.803	0.340
Hyperglycemia (*n*/%)	40 (27.40)	41 (24.56)	2.605	0.105
Hyperlipidemia (*n*/%)	39 (26.71)	40 (22.99)	4.702	0.093
LVEF (%)	40.09 ± 5.39	40.69 ± 6.31	0.185	0.854
LVEDD (mm)	51.58 ± 3.80	52.47 ± 4.04	0.452	0.663
TC (mmol/L)	4.44 ± 0.67	4.31 ± 0.61	0.351	0.715
TG (mmol/L)	1.49 ± 0.82	1.58 ± 0.73	0.152	0.638
LDL (mmol/L)	2.74 ± 0.49	2.78 ± 0.51	0.263	0.820
Number of diseased branches (*n*/%)			1.007	0.741
Single	97 (66.44)	114 (65.52)		
Double	28 (19.18)	34 (19.54)		
Triple	21 (14.38)	26 (14.94)		
SYNTAX score (*n*/%)			0.751	0.396
Grade I	68 (46.58)	81 (46.55)		
Grade II	78 (53.42)	93 (53.45)		

LVEF, left ventricular ejection fraction; LVEDD, left ventricular end-diastolic diameter; TC, total cholesterol; TG, triglyceride; LDL, low-density lipoprotein.

### 3.2 Comparison of heart rate variability indexes

**Figure 2:**
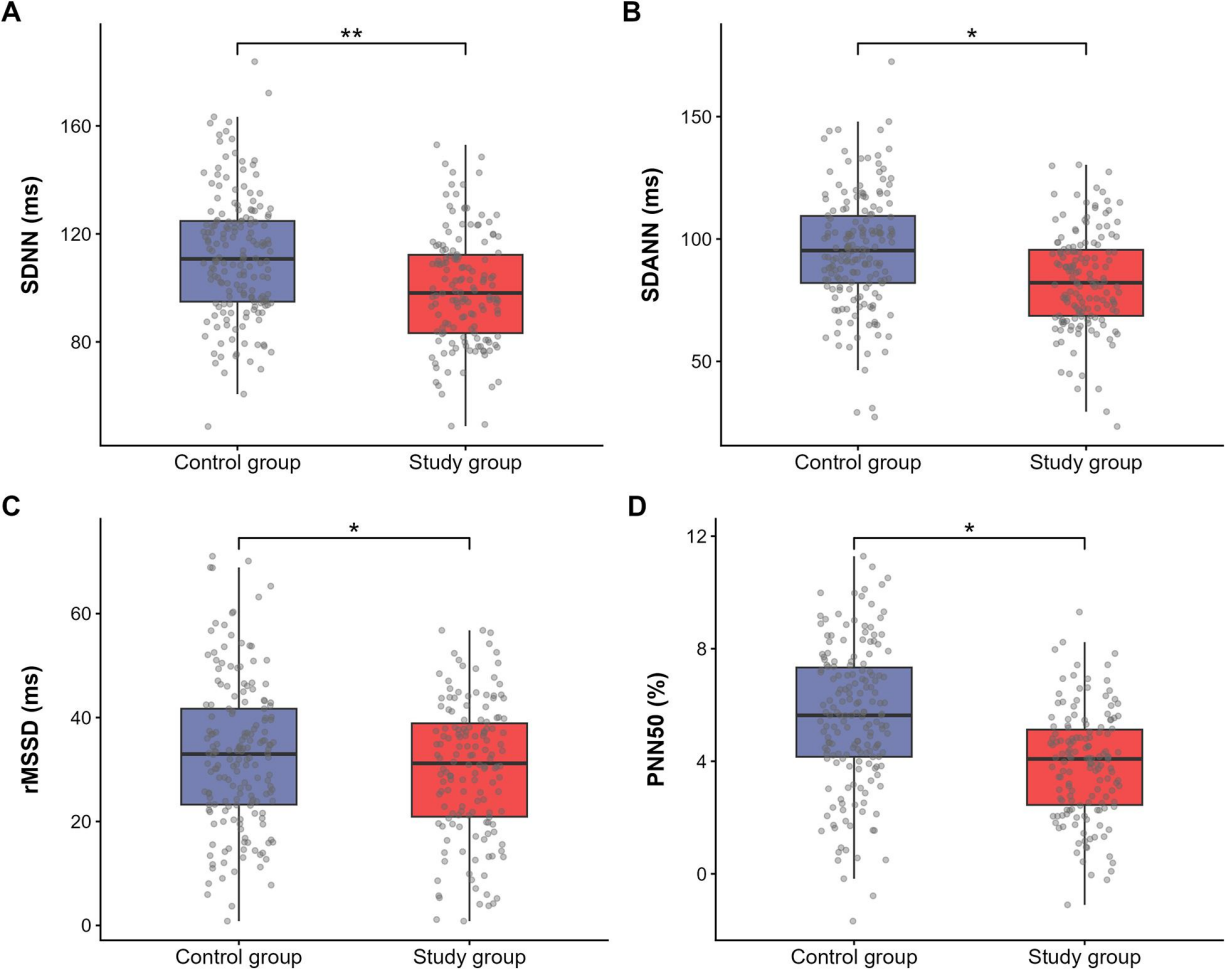
Comparison of heart rate variability (HRV) indices between the non-VA group and the VA group. **(A)** SDNN: standard deviation of normal R-R intervals; **(B)** SDANN: standard deviation of the average normal R-R intervals; **(C)** rMSSD: root mean square of successive differences; **(D)** PNN50: percentage of successive R-R intervals greater than 50 ms. Data are presented as mean ± SD, and individual data points are displayed as jittered dots to show the underlying distribution. Non-VA group, *n* = 174; VA group, *n* = 146. Differences between the two groups were analyzed using the independent-samples *t* test. *P < 0.05, **P < 0.01 compared with the non-VA group.

The comparison of HRV-related indexes between the two groups showed that the levels of SDNN (99.97 ± 20.09 vs. 113.59 ± 24.94), SDANN (80.88 ± 22.67 vs. 95.73 ± 22.46), rMSSD (30.34 ± 12.50 vs. 33.36 ± 15.23), and PNN50 (3.52 ± 1.84 vs. 5.43 ± 2.38) in the VA group were significantly lower than those in the non-VA group (*P* < 0.05). It is suggested that there are differences in autonomic nerve injury between patients with VA with CHD and patients without VA with CHD (see Table 2 and Figure 2).

**Table 2:** Comparison of HRV indices between the VA group and the non-VA group.

Index	VA group (*n* = 146)	Non-VA group (*n* = 174)	*t*	*P*
SDNN (ms)	99.97 ± 20.09	113.59 ± 24.94	10.901	0.003
SDANN (ms)	80.88 ± 22.67	95.73 ± 22.46	8.384	0.011
rMSSD (ms)	30.34 ± 12.50	33.36 ± 15.23	5.056	0.030
PNN50 (%)	3.52 ± 1.84	5.43 ± 2.38	3.484	0.028

The standard deviation of continuous normal R-R interval within 24 h (SDNN), the standard deviation of the mean value of continuous normal R-R interval every 5 min within 24 h (SDANN), the root mean square of the difference between adjacent normal RR intervals within 24 h (rMSSD), the percentage of the number of R-R interval and the average R-R interval greater than 50 ms in the total number (PNN50).

### 3.3 Correlation between heart rate variability indexes and ventricular arrhythmia occurrence

The occurrence of VA (*Y*) was used as the dependent variable, and the HRV index (SDANN: *X*1, SDANN: *X*2, rMSSD: *X*3, PNN50: *X*4) was used as the independent variable for the linear regression test. The results showed that SDANN, rMSSD, and PNN50 were correlated with VA (all *P* < 0.05). The linear equation was obtained as follows: *Y* = 1.976 – 0.006 × *X*2 – 0.009 × *X*3 – 0.007 × *X*4 (see Table 3).

**Table 3:** Linear regression analysis of the association between HRV indices and VA occurrence.

Index	*B*	95% CI	SE	Beta	*t*	*P*
Constant	1.976	1.681 to 2.270	0.148	–	12.539	0.000
SDNN (ms)	−0.004	−0.009 to 0.000	0.002	−0.249	−2.082	0.050
SDANN (ms)	−0.006	−0.011 to −0.002	0.004	−0.241	−2.381	0.021
rMSSD (ms)	−0.009	−0.015 to −0.003	0.008	−0.350	−3.030	0.013
PNN50 (%)	−0.007	−0.010 to −0.002	0.007	−0.293	−2.457	0.008

## 4 Discussion

As a common complication of CHD, VA is the result of a variety of factors, including heart disease, lifestyle, drug effects, electrolyte imbalance, and abnormal neurohumoral regulation (14). Due to an insufficient blood supply to the heart, patients with CHD can easily develop changes in myocardial electrophysiological characteristics, which can lead to VA (15). In addition, autonomic nerve dysfunction in patients with CHD, especially the decreased activity of the vagus nerve and increased sympathetic nerve activity, will further induce VA (16). Heart rate variability is an important index to evaluate the autonomic nervous function of patients with CHD. Studies have shown that the reduction of HRV is positively correlated with the risk of arrhythmia (17). Exploring the relationship between VA and HBV in CHD plays an important role in reducing the mortality of patients with CHD. This study explores and analyses the relationship between HRV and VA and provides a reference for the treatment and prevention of clinical CHD.

The results of this study found that the levels of SDNN, SDANN, rMSSD, and PNN50 in the VA group were lower than those in the non-VA group. It is suggested that there are differences in autonomic nerve injury between patients with VA with CHD and patients without VA with CHD. Time-domain HRV indices (SDANN, rMSSD, and PNN50) were used to reflect autonomic nervous system function. Among these, SDANN primarily reflects longer-term (slower) components of HRV (18). In patients with CHD, the enhancement of sympathetic nerve activity will lead to an increase in heart rate instability, which, in turn, will affect HRV indicators. This autonomic nervous imbalance is not only related to the occurrence and development of CHD but also related to the prognosis of patients. The rMSSD and PNN50 mainly reflect the rapid changes in parasympathetic nerves, especially in the case of arrhythmia, where the decrease in vagus nerve function is more significant (19, 20). Studies have shown that the occurrence of VA in patients with CHD has a significant correlation with a reduction in SDNN, SDANN, and PNN50, which is consistent with the conclusion of this study (13). The decrease in these indicators not only reflects the disorder of autonomic nervous function but may also be a good indicator for predicting the occurrence of VA.

This study further analysed the linear relationship between HRV index and VA. The results showed that SDANN, rMSSD, and PNN50 were correlated with VA, which was similar to the conclusion presented by Li et al. (21). This association may be explained by multiple pathophysiological mechanisms, including autonomic nervous system dysfunction, myocardial injury/cardiomyopathy, inflammatory responses, coronary artery disease progression, cardiac remodelling, and deterioration of cardiac function. These factors work together, resulting in a decrease in HRV indicators, thereby increasing the risk of VA (22). Studies have shown that patients with CHD exhibit an overall reduction in HRV during both daytime and nighttime. In addition, the normal circadian variation of HRV may be blunted, indicating impaired autonomic modulation across the 24-hour period (23). This autonomic imbalance is often reflected in frequency-domain measures, including higher LF power, lower HF power, and an increased LF/HF ratio (24). In addition, studies have reported that a reduction in HRV has clinical value in assessing the risk of VA and sudden cardiac death (25). However, the results of the present study showed no correlation between SDNN and VA, which may have been related to the number of patient cases, the time of the dynamic ECG, and regional differences between patients. Different monitoring times may lead to differences in results, because HRV indicators may vary over time.

This study has some limitations. Due to constraints in research time and costs, the sample size was small, and only one hospital was included in the study. The results may, therefore, be biased and lack universality. The cohort included only patients who met the predefined eligibility criteria and had complete 24 h Holter data suitable for HRV analysis; thus, the proportion of included cases among all CHD admissions during the study period could not be determined from the available dataset. In addition, validated patient-level data on VA subtype classification and baseline use of beta-blockers rate-lowering agents were unavailable, precluding subtype-specific and medication-specific counts. Second, this study was retrospective, and during data collection, memory bias may have occurred. Therefore, in our next study, we will continue to increase the sample size, extend and control the monitoring time, and consider regional differences to improve the reliability of the research results. At the same time, a prospective study will be conducted to further infer the causal relationship between HRV and VA.

## 5 Conclusion

In summary, reduced HRV was significantly associated with the occurrence of VA in patients with CHD, suggesting that HRV may be a useful indicator of VA risk stratification. The decrease in HRV reflects impaired cardiac autonomic function and may be related to an increased risk of adverse cardiovascular events in patients with CHD.

## Data Availability

The original contributions presented in the study are included in the article/Supplementary Material, further inquiries can be directed to the corresponding author.
